# Spectral Features Analysis for Print Quality Prediction in Additive Manufacturing: An Acoustics-Based Approach

**DOI:** 10.3390/s24154864

**Published:** 2024-07-26

**Authors:** Michael Olowe, Michael Ogunsanya, Brian Best, Yousef Hanif, Saurabh Bajaj, Varalakshmi Vakkalagadda, Olukayode Fatoki, Salil Desai

**Affiliations:** 1 Department of Industrial and Systems Engineering, North Carolina Agricultural and Technical State University, Greensboro, NC 27411, USA; msolowe@aggies.ncat.edu (M.O.); maogunsanya@aggies.ncat.edu (M.O.); omfatoki@aggies.ncat.edu (O.F.); 2 Center of Excellence in Product Design and Advanced Manufacturing, North Carolina Agricultural and Technical State University, Greensboro, NC 27411, USA; 3 Information Technology (IT) and Smart Manufacturing & Artificial Intelligence (SMAI), Micron Technology Virginia, 9600 Godwin Dr., Manassas, VA 20110, USA; bbest@micron.com (B.B.); yhanif@micron.com (Y.H.); sbajaj@micron.com (S.B.); varalakshmiv@micron.com (V.V.)

**Keywords:** 3D printing, acoustic data acquisition, Artificial Intelligence, quality prediction, digital signal processing, librosa, spectral feature analysis

## Abstract

Quality prediction in additive manufacturing (AM) processes is crucial, particularly in high-risk manufacturing sectors like aerospace, biomedicals, and automotive. Acoustic sensors have emerged as valuable tools for detecting variations in print patterns by analyzing signatures and extracting distinctive features. This study focuses on the collection, preprocessing, and analysis of acoustic data streams from a Fused Deposition Modeling (FDM) 3D-printed sample cube (10 mm × 10 mm × 5 mm). Time and frequency-domain features were extracted at 10-s intervals at varying layer thicknesses. The audio samples were preprocessed using the Harmonic–Percussive Source Separation (HPSS) method, and the analysis of time and frequency features was performed using the Librosa module. Feature importance analysis was conducted, and machine learning (ML) prediction was implemented using eight different classifier algorithms (K-Nearest Neighbors (KNN), Support Vector Machine (SVM), Gaussian Naive Bayes (GNB), Decision Trees (DT), Logistic Regression (LR), Random Forest (RF), Extreme Gradient Boosting (XGB), and Light Gradient Boosting Machine (LightGBM)) for the classification of print quality based on the labeled datasets. Three-dimensional-printed samples with varying layer thicknesses, representing two print quality levels, were used to generate audio samples. The extracted spectral features from these audio samples served as input variables for the supervised ML algorithms to predict print quality. The investigation revealed that the mean of the spectral flatness, spectral centroid, power spectral density, and RMS energy were the most critical acoustic features. Prediction metrics, including accuracy scores, F-1 scores, recall, precision, and ROC/AUC, were utilized to evaluate the models. The extreme gradient boosting algorithm stood out as the top model, attaining a prediction accuracy of 91.3%, precision of 88.8%, recall of 92.9%, F-1 score of 90.8%, and AUC of 96.3%. This research lays the foundation for acoustic based quality prediction and control of 3D printed parts using Fused Deposition Modeling and can be extended to other additive manufacturing techniques.

## 1. Introduction

Material extrusion is one of the most prominent AM processes utilized for several practical applications [1,2,3]. This process involves building three-dimensional structures layer-by-layer by melting filament materials in a heated extruder through the nozzle. Fused deposition modeling (FDM) is a specific type of material extrusion process that focuses on 3D printing of polymeric materials and related composite structures. FDM-printed parts have the advantages of relatively lower cost, little complexity, and faster prototyping compared to other 3D printing processes. For FDM-printed parts to be fully qualified for scalable manufacturing processes, there is a need to ensure high reliability and durability of the process and the final products. Reliable AM processes produce significantly high-quality products and help lower manufacturing costs; therefore, real-time monitoring of the AM process [4] is crucial to ensure high quality standards. In the past years, post-manufacturing techniques have been adopted to ensure that printed parts meet the required specifications, such as checking dimensional accuracy and surface roughness [5,6]. Additionally, researchers have explored image and vision-based techniques to monitor the quality of printed parts through the use of microscopes, cameras, and other sophisticated equipment [4,7,8]. However, some of these processes require human intervention, which could be time-consuming and prone to errors. In the current age of Artificial Intelligence (AI), numerous machine learning methodologies [9,10,11] have been devised to improve online monitoring procedures and guarantee process consistency [12,13,14].

The FDM set-up has several moving parts that produce acoustic waves during operations; the main sources of sound are the print bed, extruder assembly, stepper motors, filament extrusion, and the fans. Acoustics sensors, such as high-frequency microphones, can collect sound signals during the printing process, which can be further analyzed to determine print defects and underlying variations in print quality. These sensors have the advantages of lower hardware costs, much faster response, adaptable configurations, and little or no modifications to the actual AM process [15]. This approach to quality assurance (QA) in AM using acoustic signatures should lay a solid foundation for developing a framework for part qualification. The remaining sections of this paper are organized as follows: The reviews of past and existing works are highlighted in the literature review section, the unique approach that we have adopted in our research is presented, followed by results and findings. We conclude the article by discussing the summary of our findings and the key next steps.

## 2. Literature Review 

Studies have been conducted on feature extraction, process monitoring, and quality assurance in AM procedures. Bo Shen et al. [16] employed two digital microscopes for extracting relevant features from 3D-printed parts. The images obtained from the microscopes were converted into variables, which were used for testing their proposed Clustered Discriminant Regression (CDR) algorithm. The approach was found to have outperformed other conventional methods. Further, efforts were made by LK Chang et al. [17] to predict product properties in metal 3D printing by extracting layer-by-layer features using an approach called the gray-level co-occurrence matrix (GLCM), with the boosting algorithms (XGB and LightGBM) giving promising prediction results. Also, Xin Lin et al. [18] worked to improve the effectiveness of defect detection in a Selective Laser Melting (SLM) process using a fused feature-based approach in conjunction with a Support Vector Machine (SVM) algorithm to achieve an accuracy of up to 97%. 

Additionally, an online process monitoring methodology for AM processes was developed by Chenang Liu et al. [19], wherein they combined a manifold learning approach with supervised classification and regression techniques to extract features from high-dimensional sensor data with the objective of identifying AM process quality. Similarly, Zhangyue Shi [20] and his team developed a convolutional and statistical-based approach for correlating surface quality and process parameters in metal additive manufacturing, achieving classification accuracy of up to 86%. Researchers have leveraged the capabilities of sensors in AM for predicting defects and printing abnormalities. Herzog, T. et al. [21] developed an approach for detecting defects in the printed parts from the Directed Energy Deposition (DED) process by monitoring the melt pool through the use of high-speed infrared cameras. A similar approach was adopted by Grasso et al. in the selective laser melting (SLM) of zinc powder using infrared cameras for predicting abnormal melt pool conditions [22]. In addition to the melt pool monitoring, optical, thermal, and vibration sensors have been used for monitoring surface quality and defect correction in AM processes.

Despite the widespread use of vision sensors, the ease of sensor integration and calibration remains challenging. Fewer researchers have studied the use of acoustic sensing for predicting print quality and identifying defects in AM, even though equipment and set-up costs are relatively cheaper and sensor integration is much easier compared to other sensor types. S.A. Shevchik et al. [23] used acoustic emission (AE) sensors and machine learning in the powder bed AM process to classify print quality on the basis of variation in pore concentration. They attained an accuracy that was in the range of 83–89%. Using a similar approach, Hossein Taheri et al. [24] used acoustic signatures for the in situ monitoring of metal additive manufacturing process under different process conditions. His group combined acoustic sensing with K-mean clustering for classifying different process conditions. Researchers have made efforts to leverage different machine learning algorithms using supervised [25,26,27,28], unsupervised [29], and ensemble learning techniques [30] in critically analyzing AE data. 

The use of AE in Structural Health Monitoring (SHM) is becoming increasingly crucial for the study of FDM processes. Wu et al. [31] developed an online monitoring method for fused filament fabrication (FFF) by collecting AE hits data in time and frequency domains to predict failed and normal printing conditions. Z. Yang et al. [32] also developed an approach that allows for the identification of filament breakage in the FDM printing process using acoustic emission signals. Jie Liu et al. [33] proposed a method for classifying and recognizing machine states of the extruder by collecting AE data and extracting relevant features in time and frequency domains. Table 1 below adapted from PR Prem et al. [34] shows a brief overview of different machine learning algorithms applied for acoustic emission data analysis.

Our research introduces an enhanced sensor-integration approach that adopts a Linux-based system for real-time, high-efficiency data acquisition for accurate print quality prediction while minimizing data latency. Our implementation of an ML-based spectral feature extraction technique within Python 3.10.12 IDE extends beyond time and frequency-domain analyses previously conducted by Jie Liu et al. [33], which relies on vibration AE signals collected from only an FDM extruder assembly. Our approach offers more extensive insights into the behavior of acoustic signals potentially identifying failure modes within the entire AM print and improving the quality of collected acoustic signals through the use of an HPSS technique. Unlike prior research which has focused on specific aspects such as filament breakage by Z. Yang et al. [32] or extruder states by Jie Liu et al. [33], our approach aims for comprehensive quality prediction. Furthermore, while acoustic methods have been broadly applied in other AM processes like the powder bed system [23], this study provides an FDM-specific acoustic analysis for quality prediction by statistically analyzing spectral information from audio signals and its correlation with print quality layer-by-layer. Our research advances real-time process monitoring and quality prediction in AM beyond existing studies conducted by Wu et al. [31] which focuses on acoustic emission on first print layer as the foundation for its analysis of failure detection.

## 3. Methodology

### 3.1. Experimental Set-Up

**Figure 1:**
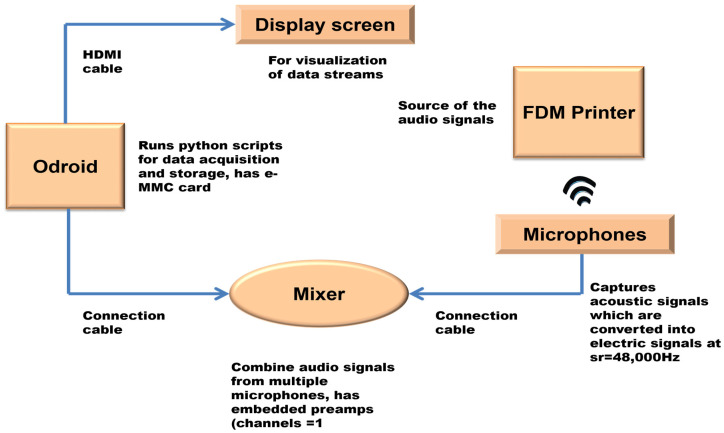
Schematic of the acoustic sensor experimental set-up.

The framework of conventional acoustic emission (AE) monitoring systems comprises AE transducers, an external or in-built preamplifier, and a data acquisition and storage system equipped with software for data visualization and analysis. Figure 1 illustrates the fundamental configuration used in the acoustic data collection set-up. Samples of cubes measuring 10 mm × 10 mm × 5 mm were fabricated using an additive manufacturing (AM) printer—Snap maker A350T 3-in-1 3D printer—which is housed in a safety transparent enclosure (Snapmaker, Shenzhen, China). Two high-frequency microphones were employed as transducers, collecting the sound waves and converting them into electrical signals during the printing process. The electrical signal is digitized by an Analog-to-Digital Converter to obtain the digital representation through sampling. A single-board computer, a 2GHz ODROID-XU4Q (Hardkernel Co., Ltd., Anyang-si, Republic of Korea) running on Ubuntu Linux 22.04 LTS (Jammy Jellyfish), was utilized for executing Python scripts and equipped with a multi-media interface card (e-MMC) of 128 GB of memory size for storing the audio data. The Odroid is a compact system that has high computational efficiency and can help with data processing and storage with minimal levels of data latency. 

The HF microphones and Odroid were connected to a mixer (Scarlett 18i20 Focusrite) (Focusrite Audio Engineering Ltd., High Wycombe, UK) embedded with a dynamic preamplifier to enhance and standardize the audio signals. Additional auxiliary equipment included a display screen for data visualization, a keyboard, connecting cables, and a mouse. The microphone placement is very crucial. The microphones 1 and 2 were placed in close proximity to the center of the build plate without any interference, at an angle of depression of 45°, and were held firmly in place by adjustable holders. The extruder and nozzle were kept at a temperature of 220 °C and the heated bed was maintained at 60 °C throughout the experiment. The filament type used is 1.75 mm PLA filament. All collected audio data was stored as a .wav file.

### 3.2. Data Acquisition 

A cube with dimensions of 10 mm × 10 mm × 5 mm was designed in SolidWorks 2023. The Luban software slices the three-dimensional model and saves it in readable format (G-code) for the Snap-maker A350T. Several samples were printed on the Snap-maker at layer thicknesses of 0.2 mm, 0.3 mm, 0.70 mm, 1.0 mm, and 1.3 mm at a constant infill density (IF) of 50% and wall thickness of 0.3 mm. The Python script was run using a PyAudio module with the channel = 1 (mono), a chunk size of 2048, and a hop length of 512. The sampling frequency of 48,000 Hz was adopted within a 16-bit audio encoding system. This sampling rate was chosen to ensure high quality of collected audio samples and prevent information loss, as stated by the Nyquist–Shannon theorem [40]. The microphone sensors collect the audio signal during printing of each sample and store it as a WAV file. Two microphones were held by adjustable holders and placed in close proximity to the printed sample. The octa-core processor of the Odroid XU-4Q is able to provide enough computational power for processing the collected audio data during the experiment, without any significant information loss. The chosen sampling rate is commonly used in professional digital audio applications.

**Figure 2:**
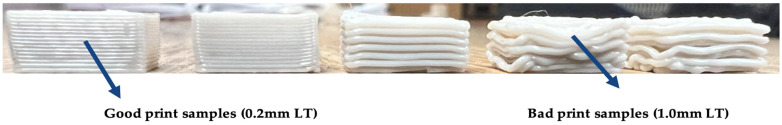
Image showing printed samples of varying layer thickness.

Based on visual inspection and expert domain knowledge, the printed samples with layer thicknesses of 0.2 mm and 0.3 mm were classified as good prints and those of 0.7 mm, 1.0 mm, and 1.3 mm as bad prints. From now on, the terms “good” and “bad” prints will be used according to the layer thicknesses provided earlier. Figure 2 below shows an image of some of the printed samples.

### 3.3. Data Preprocessing 

**Figure 3:**
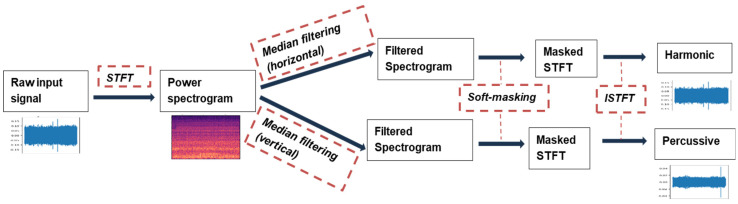
The schematic of the HPSS algorithm.

The collected audio signals were preprocessed using a denoising technique called Harmonic–Percussive Source Separation (HPSS). This approach has been described in the literature for sound source separation [41,42] and denoising. In HPSS, the harmonic segment of the sound is separated from the percussive component. As it relates to 3D printing, the harmonic segment indicates the stable, repetitive sound that emanates from the printer, such as the movement of the print platform, extruder assembly, and the stepper motors during printing. However, the percussive segments are transient audio signals such as environmental noise during the printing process. The harmonics are multiples of the fundamental frequency of the audio which are computed by converting the audio samples, from time to frequency domain through short-time Fourier transform (STFT), into spectrograms, extracting the peak frequency from the frequency bins and separating them from the lower frequency bands. The resulting sound is derived by reconstructing the signal using inverse short-time Fourier transform (ISTFT). This denoising technique has proven to be effective based on experimental observations, as denoised audio signals closely matched the resulting sound signals obtained when environmental noise around the 3D printer was recorded separately for some audio samples and the derived spectrograms were subtracted from those of the raw sound signals. External noise can significantly interfere with the quality of the audio samples by reducing the Signal-to-Noise Ratio (SNR), which makes differentiating real AM audio signals from environmental or background noise challenging. The schematic of the HPSS algorithm is given in Figure 3 below.

### 3.4. Spectral Feature Extraction

Sound signals can be analyzed statistically to extract different features. Researchers focusing on sound and music classification have utilized three methodologies: time, frequency, and time–frequency analysis. In this research, our focus is on independently extracting time-domain and frequency-domain components of AM sounds. Table 2 shows a description of the different features of the acoustic signals used in the sound experiment. The mean and variance of these spectral features were used within the machine learning models to identify good and bad 3D printed parts. As an example, higher ZCR suggests more rapid changes in the movement of the print head or excessive vibration of the print bed or frame, potentially relating to surface roughness or dimensional inaccuracies. Thus, the bad part had higher mean (0.105) and variance (7.83 × 10^−4^) ZCR as compared to the good part mean (0.09) and variance (7.1 × 10^−4^). Similarly, higher RMS energy levels indicate that there is over-extrusion or higher heat generation, which could affect the quality of the printed sample, and were reported for a bad print (mean: 0.0102 and variance: 1.45 × 10^−5^) as compared to a good print (mean: 0.01 and variance: 1.53 × 10^−5^). A flatter spectrum usually points more towards consistent printing conditions for a good print (mean: 0.023 and variance: 7.35 × 10^−4^), while peaks could indicate printing issues for a bad print (mean: 0.0275 and variance: 1.186 × 10^−3^). 

The mean and standard deviation of the time and frequency-domain features were extracted from 10-s segments of the audio samples corresponding to each layer thickness setting. The printing times for the 0.2 mm, 0.3 mm, 0.7 mm, 1.0 mm, and 1.3 mm LT are 300 s, 210 s, 120 s, 80 s, and 61 s, respectively. The computation of these features was performed in a Python package called Librosa library. This Python module is very robust and widely used for audio and music analysis.

### 3.5. Machine Learning

**Figure 4:**
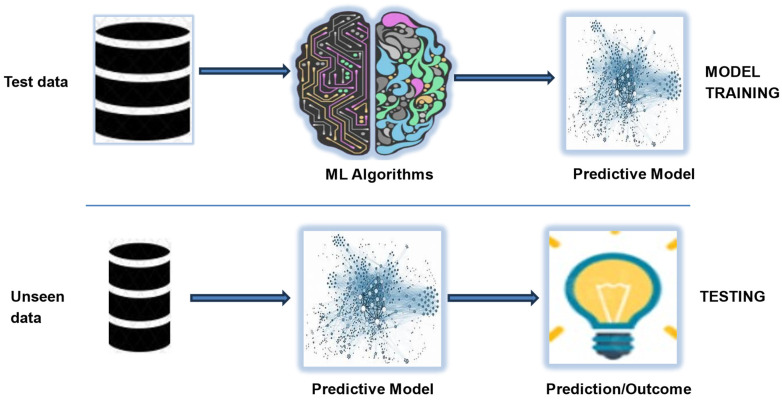
Typical machine learning steps for classification.

A Schematic of a typical supervised machine learning project is shown in Figure 4. Our methodology includes normalizing the dataset using the “Standard Scaler”, determining feature ranking and importance with the Extra Tree Classifier, and carrying out model training, validation, and performance assessment. Standard scaler helps to scale the features to fit a standard distribution and ensures that no feature significantly influences the model performance as a result of the differences in scales and units of the individual features. Machine learning algorithms such as Logistic regression, Support Vector Machines, and Gaussian Naïve Bayes perform better when given datasets that are standardized. Additionally, ExtraTree Classifier is a chosen candidate because it is an ensemble technique that constructs decision trees in parallel and can potentially speed up model training compared to other feature selection techniques [49]. This technique randomizes data subsets into multiple decision trees, which can reduce bias and provide a more comprehensive evaluation of feature importance [50].

Eight different ML classifiers were employed for predicting print quality based on the collected temporal and spectral data of the printed samples. The supervised learning technique was employed, as the output variables were labeled (print quality) and coded as “1” for good print and “0” for bad print. The classifiers and a brief description are presented below. See Table 3.

## 4. Results and Discussion

**Figure 5:**
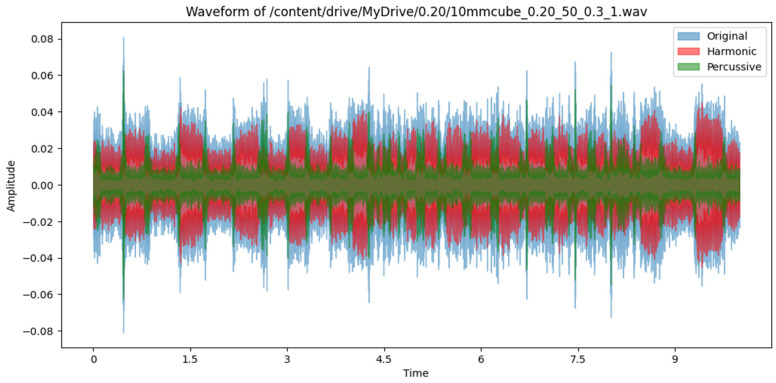
Visual representation of the harmonic and percussive segments of an AM sound signal.

A snapshot of the audio preprocessing that was conducted in this research using the HPSS is presented below. In Figure 5 below, the waveform of a 10-s-long sample audio of layer thickness of 0.2 mm, an infill density of 50%, and a wall thickness of 0.3 mm is shown. The harmonic and percussive components of the signal were separated using the librosa.effects.hpss module.

**Figure 6:**
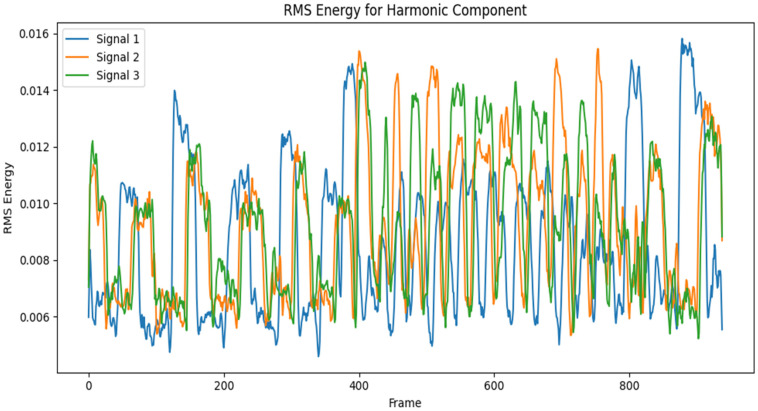
RMS energy vs frame index of a 10-s audio segment of AM sound signals.

**Figure 7:**
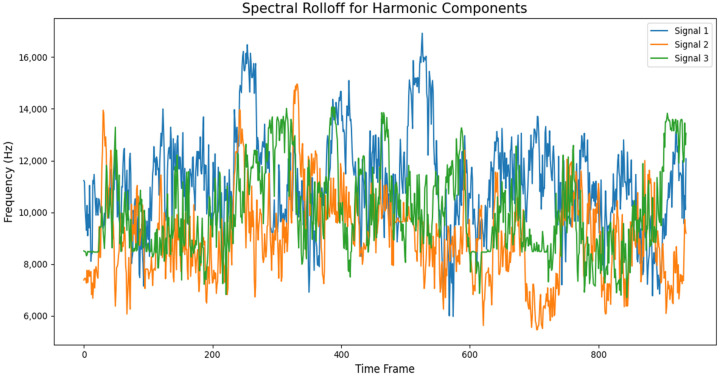
Spectral roll-off vs frame index of a 10-s audio segment of AM sound signals.

An analysis of the time and frequency-domain features of a 10-s audio segment of different layer thicknesses is presented in Figure 6 and Figure 7. As can be seen in the graphs, there were distinctive behavioral patterns exhibited by the different audio samples when computing the RMSE and SR against corresponding frame indices. Signal 1 represents a good print segment, while signals 2 and 3 are bad print segments. Signal 1 is an audio segment from a 0.2 mm layer thickness sample, while signals 2 and 3 were samples from layer thicknesses of 0.7 mm and 1.3 mm, respectively, collected at the same time stamp. The RMSE and frequency bands of the corresponding signals varied across the specified time window.

**Figure 8:**
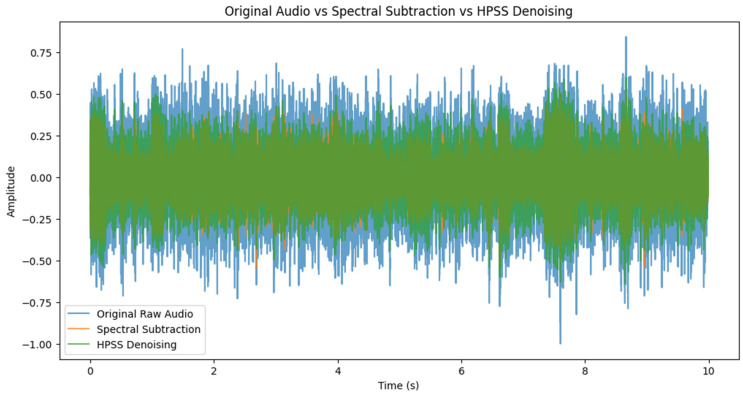
Amplitude vs time comparison of raw audio and denoised audio (spectral subtraction and HPSS).

This section presents the findings and insights gained from analyzing the time and frequency-domain features extracted from the audio samples collected during the 3D printing process. It also demonstrates the effectiveness of using ML algorithms to predict quality outcomes based on extracted features. The feature importance analysis was carried out on the collected dataset to provide insights into the most relevant audio features using the Extra Trees classifier algorithm. The spectral relationship between all the identified features was visually shown in the form of a correlation matrix. The standardized and cleaned dataset is trained on eight classification algorithms, including ensemble and boosting techniques, and the performance analysis was performed by measuring and comparing the prediction, precision, recall, and F-1 score on a test dataset which represents 20% of the entire dataset. The collected audio signals were preprocessed using a denoising technique called Harmonic–Percussive Source Separation (HPSS), a technique that separates audio signal into stable printer sounds (harmonics) and transient environmental noises (percussives). The time-dependent audio signal is first converted into a time-frequency component using a technique called short-time Fourier transform. The resulting time–frequency-domain signal is then smoothened in the horizontal and vertical direction using defined median filters. Soft masks are created based on the filtered vertical and horizontal spectrograms which determine what proportion of each frequency bin belongs to either the harmonic or percussive segment. This HPSS algorithm we employed was executed in Python using the librosa.effects.hpss module, and a sample result has been presented in Figure 5. We validated the effectiveness of HPSS by comparing its outcome with the resulting sound data obtained when we discounted environmental sound from recorded raw AM sound just by mere spectral subtraction of environmental sound from recorded AM sound. In all, the technique enhanced the quality of printer sounds while reducing background interference. In Figure 8 below, the amplitude of a sample raw AM audio signal is plotted visually against cleaned signals using direct subtraction of manually recorded environment noise (AM printer in non-operational mode) and applying the HPSS method described in Section 3.3 on the actual raw AM sound. It can be seen from the plot that the amplitude envelope of the denoised signal was significantly lower compared to the raw AM sound. Additionally, the amplitude of the denoised audio signals showed consistently similar patterns in the audio spectrum.

Precision measures the proportion of correctly predicted good print segments (true positives) out of all the prints predicted as good (true positives + false positives). Recall, also known as sensitivity or true positive rate, measures the proportion of actual good prints (true positives) that are correctly predicted as good by the model. Accuracy measures the overall correctness of the model’s predictions, considering both good and bad print segments. The F-1 score is a performance metric that considers the weighted average of precision and recall. Additionally, the area-under-the-curve (AUC) was measured using the ROC curves and precision–recall curves of all the eight classifier algorithms to further understand the classification performance of the machine learning algorithms. The ROC curve plots the recall versus the false positive rate (bad audio segments wrongly classified as good audio segments), while the precision–recall curve graphically shows the trade-off between precision and recall. 

Preliminary analysis was initially carried out to determine the optimal number of features to be used for training the ML algorithms as presented in Table 4. It is evident that all features need to be considered for higher accuracy. The corresponding highest-performing algorithm was noted for different feature considerations. The removal of highly correlated features resulted in lower accuracy indicating that considering all features provided better discriminating power. Furthermore, the parameters of the models were optimized to obtain the best results. This includes Logistic Regression (penalty= ‘l2′, max_iter = 100, random_state = None), Gaussian Naïve Bayes (var_smoothing = 0.000000001), K-Nearest Neighbors (n_neighbors = 5), Support Vector Machine (C = 1, gamma = scale), Decision Trees (criterion = gini, min_samples_split = 2), Random Forest (n_estimators = 100, bootstrap = True), XGBoost (learning rate = 0.3, n_estimators = 100), and LightGBM (n_estimators = 100, learning rate = 0.1).

**Figure 9:**
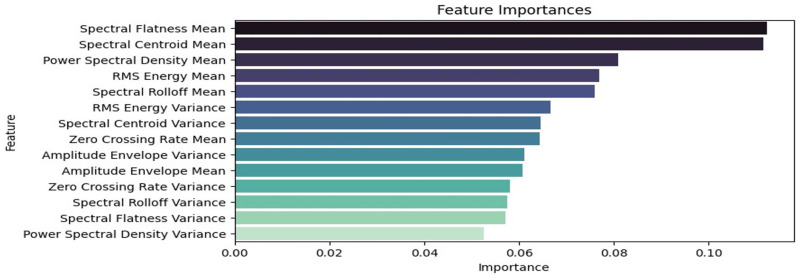
Feature ranking of the spectral features.

**Figure 10:**
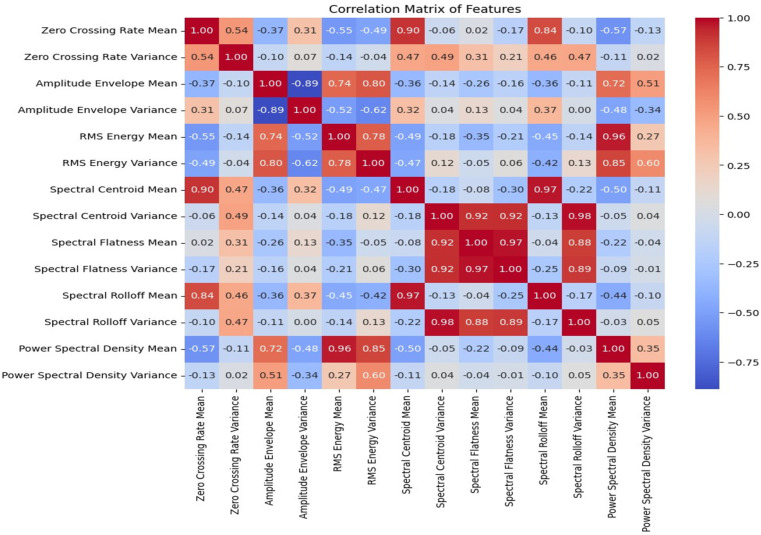
Correlation matrix of the spectral features.

Figure 9 depicts the feature importance analysis based on all the features extracted from the audio samples. It can be deduced that the mean of the spectral flatness, spectral centroid, power spectral density, and root mean square energy were the most relevant features of the audio samples. Figure 10 shows the correlation matrix with high correlations between the mean of the spectral centroid and that of the spectral roll-off, and the same is shown for the average power spectral density and that of the RMS energy. The variance of the power spectral density was the least important feature based on the feature ranking. 

**Figure 11:**
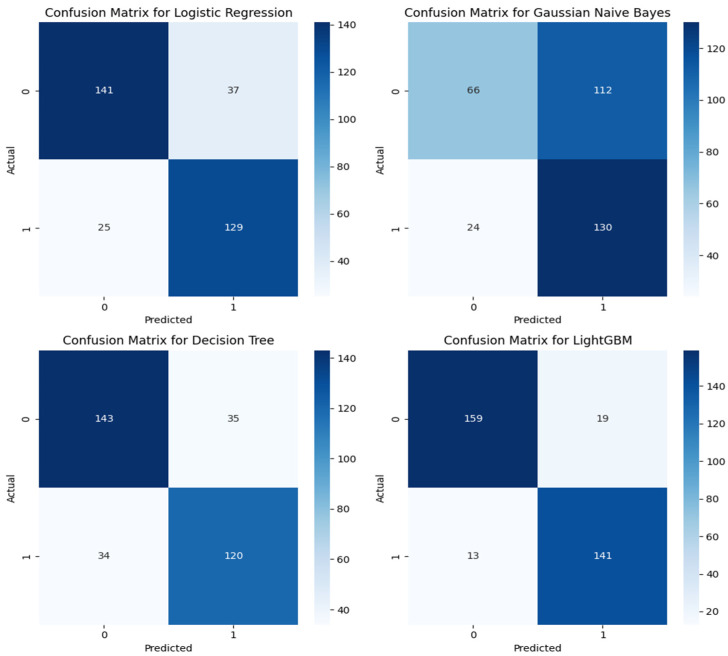
Confusion matrix of the LR, GNB, DT, and LightGBM algorithms.

**Figure 12:**
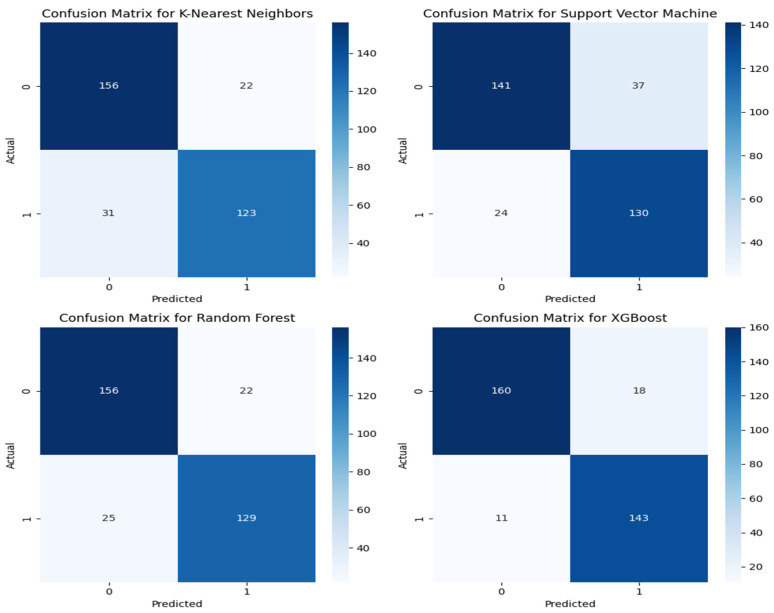
Confusion matrix of the KNN, SVM, RF, and XGB algorithms.

Figure 11 and Figure 12 below show the confusion matrix for the eight classification algorithms. The XGB classifier, a boosting algorithm, gave the highest prediction accuracy of 91.3% and the lowest accuracy for the Gaussian Naïve Bayes (GNB) at 59.0%. The precision, recall, and F-1 scores for the XGB were 88.8%, 92.9%, and 90.8%, respectively. From the confusion matrix of the best model, 303 audio segments were correctly classified, while 29 instances were misclassified by the model based on the 332 audio segments used for model validation. Of the 161 good print predictions, 143 audio segments were correctly classified. In addition, out of the actual 154 good audio segments, 11 were wrongly classified. In the GNB algorithm (the least effective model), it misclassified 136 audio instances and correctly predicted 196 instances. However, the recall was as high as 84.4% because there were only 18 actual good audio segments wrongly classified by the model. Ensemble and boosting algorithms outperformed all the other models.

**Figure 13:**
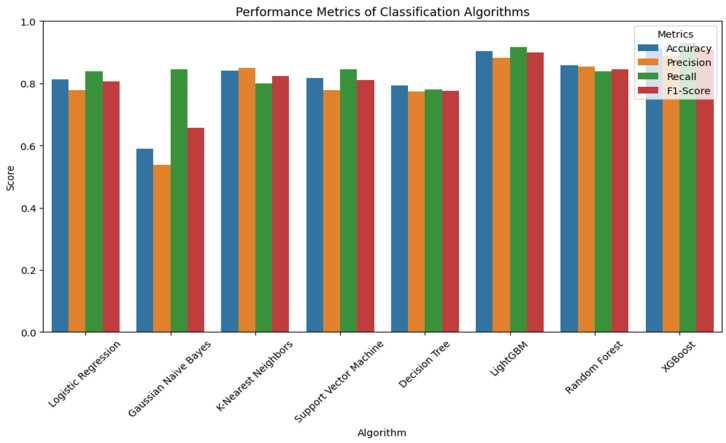
The bar chart of the performance metrics for all the eight classifiers.

Figure 13 shows the performance of all eight models. All the classification algorithms produced prediction accuracies above 80% except for DT and GNB. The ensemble models (LightGBM, RF, and XGBoost) produced high values across all the performance metrics. Additionally, the boosting algorithms (XGB and LightGBM) produced very similar results.

**Figure 14:**
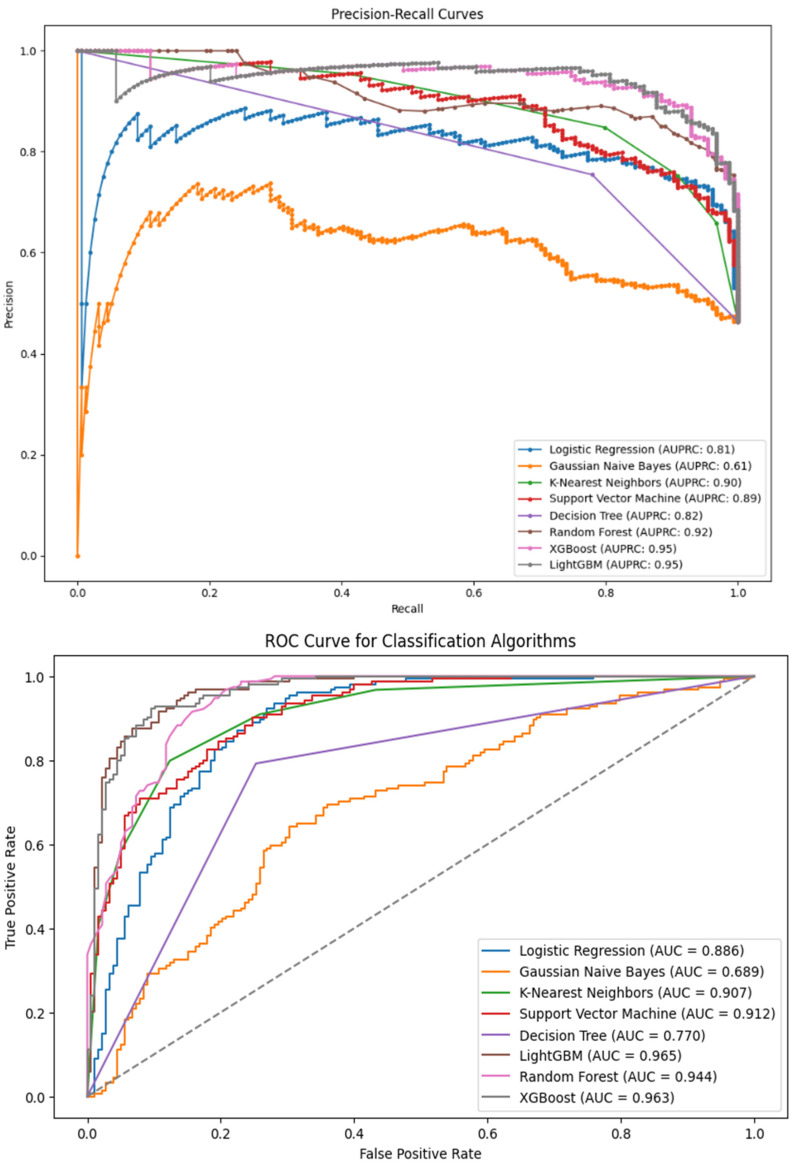
AUCROC and AUPRC for the eight classification algorithms.

Figure 14 presents the AUC of the ROC and the precision–recall for all the machine learning models. The AUC-ROC and AUPRC of 96.3% and 95.0% were obtained for the XGB, respectively. This shows consistently high values as compared with the model’s accuracy. AUC-ROC and AUPRC offer aggregate measures of the model’s performance across different thresholds.

The training time for each of the classification algorithms is presented in Table 5. The Gaussian Naïve Bayes had the lowest average training time of 2.2 ms. The relatively short training duration for all the models can be attributed to the parallel processing capability of the A100 GPU hardware accelerator utilized on Google Colab, the environment on which the Python code was run. The scaling or standardization of the spectral features before training also helped in speeding up model convergence. These faster training times provide the ability of the algorithms to be implemented on real-time acoustic datasets for practical applications in 3D printing. 

HPSS effectiveness is highlighted by its ability to specifically target and separate percussive noise components from harmonic printer operation sounds. By focusing on this separation through STFT and reconstruction through ISFT. This denoising technique has proven to be effective based on experimental observations, as denoised audio signals closely matched the resulting sound signals obtained when environmental noise around the 3D printer was recorded separately for some audio samples and the derived spectrograms were subtracted from those of the raw sound signals. Experimental validation supports HPSS’s effectiveness by demonstrating significant noise reduction and improvement in SNR, ensuring that the cleaned audio signals better reflect the operational state of the 3D printer without the interference of environmental noise. This makes HPSS a tailored and effective solution for cleaning audio data in the specific application context of 3D printing. 

The methodology adopted in the research for the machine learning predictive models holds significant promise for advancing quality assurance in Fused Deposition Modeling and other 3D-printing processes. The outcome of variation in layer thicknesses provides an important marker to predict the integrity and dimensional stability of the 3D-printed parts. This research showed that by exploring the time and frequency features of audio signatures using advanced DAQ systems for data collection and analysis, ML models can accurately classify the quality of 3D-printed parts based on process parameters. In this study, it was validated experimentally that 3D-printed samples of 10 mm × 10 mm × 5 mm cubes of layer thicknesses of 0.2 mm and 0.3 mm gave high-quality outputs and were labeled as good samples. However, samples with layer thicknesses of 0.7 mm, 1.0 mm, and 1.3 mm gave unacceptable structures and were labeled bad samples. The best-performing model, XGBoost (XGB), achieved a 91% prediction accuracy, demonstrating its effectiveness in discriminating audio samples based on observed layer thicknesses and subsequent print quality.

By integrating these models into the quality control framework of FDM and other 3D-printing processes, manufacturers can monitor and detect anomalies in process parameters such as layer thickness in real time. Thereby, it enables them to make informed decisions about print quality and take corrective action when necessary. This real-time feedback can help optimize printing parameters, reduce material waste, and improve the overall quality and consistency of 3D-printed parts. Furthermore, the application of these predictive models can pave the way for the development of closed-loop control systems and digital twins for additive manufacturing processes.

## 5. Conclusions

This research demonstrates the feasibility of using acoustic data for quality prediction and control in 3D printing, specifically in Fused Deposition Modeling (FDM). This study analyzed seven audio features in time and frequency space and presented a framework for print quality prediction by combining machine learning with advanced digital signal processing. Feature importance analysis showed that the mean of the spectral flatness was the most prominent feature. Furthermore, the findings suggest that acoustic sensing can be valuable for real-time monitoring and quality control in additive manufacturing processes. Ensemble models (XGB, RF, and LightGBM) showed high accuracy in classifying the audio segments of varying print quality. Future research can expand this approach to other additive manufacturing techniques and further refine the ML models for improved prediction accuracy. In addition to this, the scope of the research should extend beyond binary classification, and multi-class classification should be employed to accurately predict different bad print scenarios. Acoustic-based quality prediction has the potential to enhance manufacturing efficiency and product quality across various industries and should be widely embraced.

## Figures and Tables

**Table 1:** ML Algorithms applied for acoustic emission data analysis.

S/N	Algorithm Name	Method	Acoustics Applications
1	Principal Component Analysis [35]	Preserves important information while reducing the dimensionality of the dataset	Creates clusters of AE events to identify similar patterns
2	K-means Clustering [36]	Partitions data points into groups with minimum variance within each group or cluster	For identifying different types of acoustic emissions
3	Convolutional/Deep Neural Networks [37]	Uses interconnected layers with different activation functions to extract complex features	Feature extraction, handles complex AE dataset
4	Recurrent Neural Network/Long Short-Term Memory [38]	Uses sequential and memory functions to process sequential tasks	For identifying abnormalities in temporal patterns
5	Isolation Forest/One-Class SVM [39]	Creates decision trees with much lower instances in isolated partitions	For defect detection in AM processes
6	Ensemble Methods /Random Forest [30]	Aggregates predictions from multiple classes of models	Better prediction accuracy, minimizes overfitting

**Table 2:** Time and frequency-domain features of the acoustic signals.

Feature	Definition	Domain	Mathematical Representation
Zero Crossing Rate (ZCR) [43]	Number of times the waveform changes sign in a window.	Time	${ZCR}_{t}=\frac{1}{2}\;\sum_{k=t.K}^{\left(t+1\right).K-1}\left|sgn\left(s_{k}\right)-sgn\left(s_{k+1}\right)\right|$
			- sgn: sign of function (+1, −1, or 0)
Amplitude Envelope(AE) [44]	AE indicates how the energy of the signal fluctuates over time and shows the magnitude of variations directly.	Time	${AE}_{t}=\max\left(s_{\left(k\right)}\left[t.K,\left(t+1\right).K-1\right]\right)$
			- ${AE}_{t}$: AE at kth frame *t* - $s_{\left(k\right)}$: amplitude of sample - *K*: number of samples in a frame
Root Mean Squared Energy (RMSE) [45]	Root mean square of all samples in a frame. It is an indication of loudness.	Time	${RMS}_{t}=\sqrt{\frac{1}{K}}\sum_{K=t.K}^{\left(t+1\right).K-1}s_{\left(k\right)}^{2}$
Spectral Centroid (SC) [46]	It is the center of mass of the magnitude spectrum, which is determined by calculating the weighted mean of all frequencies.	Frequency	${SC}_{t}=\frac{\sum_{n=1}^{N}m_{t(n)}.n}{\sum_{n=1}^{N}m_{t(n)}}$
Spectral Flatness (SF) [47]	The geometric mean divided by the arithmetic mean of the spectra: it determines how much of a sound is noise-like versus tone-like.	Frequency	${SF}_{t}=\frac{{\left(\prod_{n=1}^{N}m_{t(n)}\right)}^{\frac{1}{n}}}{\frac{1}{n}\sum_{n=1}^{N}m_{t(n)}}$
Spectral Roll-off (SR) [15]	Fraction of bins in the power spectrum at which 85% of the power is at lower frequencies.	Frequency	${SR}_{t}=i\;s.t.\sum_{n=1}^{i}\left|m_{t(n)}\right|=\eta \sum_{n=1}^{N}\left|m_{t(n)}\right|$
Power Spectral Density (PSD) [48]	Estimates the distribution of a signal’s strength across a frequency spectrum.	Frequency	${PSD}_{dB}=10\log_{10}\left(PSD\right)$

**Table 3:** ML algorithms implemented and brief description.

Classifier	Description
Decision Tree (DT)	Decision tree is a graph to represent choices and their results in the form of a tree. The nodes in the graph represent an event or choice and the edges of the graph represent the decision rules or conditions. Each tree consists of nodes and branches. Each node represents attributes in a group that is to be classified, and each branch represents a value that the node can take.
K-Nearest Neighbors (KNN)	KNN uses data and classifies new data points based on similarity measures (e.g., Euclidean distance function). Classification is computed from a simple majority vote of the K-Nearest Neighbors of each point. KNN can be used both for classification and regression.
Random Forest (RF)	It is well known as an ensemble classification technique that uses parallel ensembling to fit several decision tree classifiers on different dataset sub-samples and uses majority voting for the outcome.
Gaussian Naive Bayes (GNB)	The naive Bayes algorithm is based on Bayes’ theorem with the assumption of independence between each pair of features. It works well and can be used for both binary and multi-class categories in many real-world situations.
Extreme Gradient Boosting (XGB)	Gradient Boosting, like Random Forests above, is an ensemble learning algorithm that generates a final model based on a series of individual models, typically decision trees. The gradient is used to minimize the loss function, similar to how neural networks work.
Logistic Regression (LR)	Logistic regression typically uses a logistic function to estimate the probabilities, which is also referred to as the mathematically defined sigmoid function.
Support Vector Machine (SVM)	A support vector machine constructs a hyperplane or set of hyperplanes which has the greatest distance from the nearest training data points in any class. It is effective in high-dimensional spaces and can behave differently based on different mathematical functions (kernel).
Light Gradient Boosting Machine (LightGBM)	It is a variant of the gradient boosting algorithm, which uses multiple sets of decision trees to create a strong predictive model. The algorithm iteratively trains DTs to minimize the loss function by trying to improve on the mistakes made by the previous trees.

**Table 4:** Feature importance analysis.

	Accuracy	Precision	Recall	F1 Score	Best Algorithm
All features	91.2	88.8	92.9	90.8	XGB
Top six features	84.3	81.4	85.7	84.2	LightGBM
Top eight features	86.1	84.2	86.4	85.3	LightGBM
Removing two highly correlated	87	86.1	84.4	85.2	Random Forest
Removing four highly correlated	88.9	87.7	88.3	88	LightGBM
PCA- 8	82.5	83.3	77.9	80.5	KNN

**Table 5:** Average training time of the classification algorithms.

Algorithm	Avg. Training Time (ms)
Logistic Regression	25.7
Gaussian Naïve Bayes	2.2
K-Nearest Neighbors	3.7
Support Vector Machines	324.2
Decision Tree	17.1
Random Forest	448.5
XGBoost	194.4
LightGBM	167.1

## Data Availability

The data presented in this study are available on request from the corresponding author.
